# Barriers to publishing in biomedical journals perceived by a sample of French researchers: results of the DIAzePAM study

**DOI:** 10.1186/s12874-017-0371-z

**Published:** 2017-07-10

**Authors:** Martin Duracinsky, Christophe Lalanne, Laurence Rous, Aichata Fofana Dara, Lesya Baudoin, Claire Pellet, Alexandre Descamps, Fabienne Péretz, Olivier Chassany

**Affiliations:** 1 Département de Médecine Interne et d’Immunologie Clinique, Hôpital Bicêtre, AP-HP, Paris, France; 2 Unité de Recherche Clinique en Economie de la Santé, URC ECO, Hôpital Fernand-Widal, AP-HP, Paris, France; 3 Patient-Centered Outcomes Research, EA 7334 REMES, Université Paris-Diderot, Sorbonne Paris Cité, Paris, France; 4 Abelia Science, Saint-Georges-sur-Baulche, France; 5 Département de la Recherche Clinique et du Développement (DRCD), Hôpital Saint-Louis, AP-HP, Paris, France

**Keywords:** Survey and questionnaire, Hospitals, University, France, publishing, Medical writing, job satisfaction, English, Difficulties, Needs, Financial support

## Abstract

**Background:**

As publishing is essential but competitive for researchers, difficulties in writing and submitting medical articles to biomedical journals are disabling. The DIAzePAM (*Difficultés des Auteurs à la Publication d’Articles Médicaux*) survey aimed to assess the difficulties experienced by researchers in the AP-HP (*Assistance Publique – Hôpitaux de Paris*, i.e., Paris Hospitals Board, France), the largest public health institution in Europe, when preparing articles for biomedical journals. The survey also aimed to assess researchers’ satisfaction and perceived needs.

**Methods:**

A 39-item electronic questionnaire based on qualitative interviews was addressed by e-mail to all researchers registered in the AP-HP SIGAPS (*Système d’Interrogation, de Gestion et d’Analyse des Publications Scientifiques*) bibliometric database.

**Results:**

Between 28 May and 15 June 2015, 7766 researchers should have received and read the e-mail, and 1191 anonymously completed the questionnaire (<45 years of age: 63%; women: 55%; physician: 81%; with PhD or *Habilitation à Diriger des recherches*––accreditation to direct research––: 45%). 94% of respondents had published at least one article in the previous 2 years. 76% of respondents felt they were not publishing enough, mainly because of lack of time to write (79%) or submit (27%), limited skills in English (40%) or in writing (32%), and difficulty in starting writing (35%). 87% of respondents would accept technical support, especially in English reediting (79%), critical reediting (63%), formatting (52%), and/or writing (41%), to save time (92%) and increase high-impact-factor journal submission and acceptance (75%). 79% of respondents would appreciate funding support for their future publications, for English reediting (56%), medical writing (21%), or publication (38%) fees. They considered that this funding support could be covered by AP-HP (73%) and/or by the added financial value obtained by their department from previous publications (56%).

**Conclusions:**

The DIAzePAM survey highlights difficulties experienced by researchers preparing articles for biomedical journals, and details room for improvement.

**Electronic supplementary material:**

The online version of this article (doi:10.1186/s12874-017-0371-z) contains supplementary material, which is available to authorized users.

## Background

The scientific objectives of medical publishing are numerous: sharing results and thus helping science to progress, optimizing patient management, benefitting from the exchange and ideas with other researchers, and becoming part of the scientific community (unread is unknown) [1, 2]. Indeed, “without publication research is sterile” [3]. In addition, publishing may impact career and fund medical research. In France, SIGAPS (*Système d’Interrogation, de Gestion et d’Analyse des Publications Scientifiques*) is a bibliometric score assigned to researchers according to the number of articles they published, their place in the author lists, and the impact factor of the journal within their field [4–6]. SIGAPS is one of the most important elements of the MERRI (*Missions d’Enseignement, de Recherche, de Référence et d’Innovation*) financial allocation system, partly dedicated to research funding in public hospitals.

Publishing is not an easy task. Firstly, it is an integral component of a research process, with its own fundamental, unavoidable rules. Medical writing is subject to the strict criteria used by journal peer-review committees, depending on the type of publication and journal (impact factor, degree of specialization, timeliness, etc.) [7]. In a concern for quality, standardization and transparency, specific guides have been developed for each type of publication, such as CONSORT for randomized controlled trials, and are widely adopted by journals [8]. In addition to these recommendations, each journal has its own Instructions for Authors [9, 10]. The publication requirements are getting more and more stringent: e.g., mandatory trial preregistration [11, 12]; institutional review board approval for all kinds of research, including retrospective observational studies. Although there is some improvement in reporting authors’ guidance in journals, these recommendations and requirements are little known and complicate the publication process [13]. Secondly, it is a field of international competition. Due to their international dimension, publications are mostly in English, whereas not every researcher or practitioner is a native English-speaker [14]. In addition, positive results that immediately change clinical practice or are on a popular topic are easier to publish than other results [15]. Trial non-publication is frequent (1 in 3 completed surgical randomized controlled trials is unpublished [16]) and represents a waste of research resources. It leads to hidden trial data and publication bias, which is deleterious as the reported magnitude of treatment effects is generally overestimated, and raises ethical concerns for patients who participated in unpublished studies [17–19].

The DIAzePAM (*Difficultés des Auteurs à la Publication d’Articles Médicaux*) survey was performed to assess the difficulties experienced by researchers in the Paris public-sector hospitals board (*Assistance Publique – Hôpitaux de Paris*: AP-HP, France) who publish or intend to publish, and to evaluate their publication satisfaction and perceived needs. AP-HP is the largest public health institution in Europe, including 38 university hospitals in Paris and its suburbs (www.aphp.fr). AP-HP doctors are strongly involved in research and training, and co-author more than 9000 articles per year in PubMed-referenced journals (i.e., 9119 articles in 2015 [20]).

## Methods

DIAzePAM is a cross-sectional study. The Independent Ethics Committee (*Comité de Protection des Personnes Ile de France IV* – Institutional Review Board – n° 00003835) confirmed that this study is observational, and that it fulfills current regulatory and ethical obligations.

The DIAzePAM questionnaire was based on literature analysis and face-to-face or phone interviews with 11 doctors (clinicians, radiologists, biologists) working in AP-HP. The authors of the questionnaire had expertise in qualitative research (MD, OC) [21, 22] and in medical writing (MD, OC, FP). The prototype electronic questionnaire was tested on 11 AP-HP medical doctors to check acceptability and comprehensiveness. The final DIAzePAM questionnaire (see Additional file 1) included items measuring medical writing experience, difficulties encountered, respondent’s position with regard to the publication of articles, and need for external support as well as non-identifying sociodemographic and occupational characteristics. At the end of the questionnaire, each respondent could add a free comment; these comments were analyzed, manually sorted according to keywords (e.g., English, medical writing, statistics, time, money or congratulations), and grouped thematically by 2 independent reviewers (AFD, LR).

An e-mail request to participate in the anonymous survey was sent to all AP-HP researchers registered in the SIGAPS database (*N* = 8186). Although some of the authors of this article (MD, OC) could receive the questionnaire, they did not participate in the study. This short e-mail included the title of the study, its objectives, the target population (AP-HP professionals who publish or intend to publish), an electronic link to the DIAzePAM questionnaire, and an estimation of the completion time (< 5 min). Researchers were also informed that results would be published. No compensation was offered for participation. LimeSurvey®, an online survey tool, was used to create the DIAzePAM questionnaire, conduct the survey, and perform the first analysis of the results. Its functioning was tested on major web browsers before the start of the survey. The questionnaire remained online for 3 weeks (from 28 May to 18 June 2015). Two reminder e-mails were sent at 1-week intervals.

Only fully completed questionnaires could be submitted and were thus analyzed. For each answer choice, raw number and percentage (versus the total number of people who gave answer to the question) were automatically described by the survey software. Pearson (categorical variables with unordered categories), and proportional odds-ratio model likelihood ratio (categorical variables with ordered response levels) chi-square tests were used to assess correlation between factors associated with difficulty in writing (i.e., limited skills in English, limited skills in writing, and difficulty in starting writing) [23, 24]. Analysis was performed using R software package.

## Results

On 28 May 2015, the e-mail request was sent to 8186 researchers; 420 (5%) e-mails could not be delivered as 73 inboxes were overloaded, and 347 e-mail addresses no longer existed. Finally, 1191 (15%) of the 7766 researchers who have possibly received the e-mail request completed the DIAzePAM questionnaire.

### Respondents and previous publications

The main sociodemographic and occupational characteristics of respondents are presented in Table 1.

**Table 1:** Sociodemographic and occupational characteristics of respondents (*N* = 1191)

	N	(%)^a^
Gender		
Women	650	(55%)
Men	541	(45%)
Age (years)		
< 35	320	(27%)
35–45	426	(36%)
45–55	269	(23%)
55–65	158	(13%)
≥ 65	18	(2%)
Occupation		
Physician	969	(81%)
Pharmacist	102	(9%)
Biologist	91	(8%)
Odontologist	13	(1%)
Other (geneticist, other unspecified)	16	(1%)
Position		
Hospital practitioner	602	(51%)
Senior registrar, hospital university assistant	245	(21%)
University professor-Hospital practitioner	172	(14%)
University assistant professor-Hospital practitioner	128	(11%)
Other	44	(4%)
Degrees^b^		
PhD	496	(42%)
Habilitation à Diriger des recherches (HDR)	241	(20%)
No PhD and no HDR	659	(55%)

Habilitation à Diriger des Recherches (HDR): French accreditation to direct research

^a^Rounded values; ^b^More than 1 answer possible

**Fig. 1:**
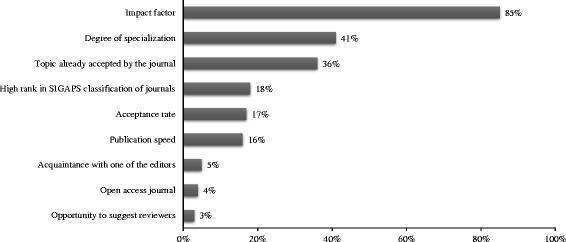
Respondents’ main reasons for choosing the journal (%). *N* = 1191; more than 1 answer possible

Regarding previous publications, 94% (*N* = 1123) of respondents had been an author of at least one article in the previous 2 years: 65% at least once as first author, and 39% as last author. 73% of authors stated that they actually participated in the writing and/or submission of their articles while 19% reported having been only investigators and 9% that their contribution has been minimal. Impact factor was the main journal selection criterion for 85% of authors. The other criteria are presented in Fig. 1.

Sixty percent of authors reported having had difficulties in writing the discussion section of their recent articles, and 28% having called upon additional support (translator: 61%; medical writer: 26%; non-author colleague: 23%). 77% of authors who called upon additional support versus 66% of authors who did not call upon additional support published at least once as first author in the previous 2 years (*P* = 0.004). The person who helped was paid in 61% of cases.

### Perceived barriers to publication

**Fig. 2:**
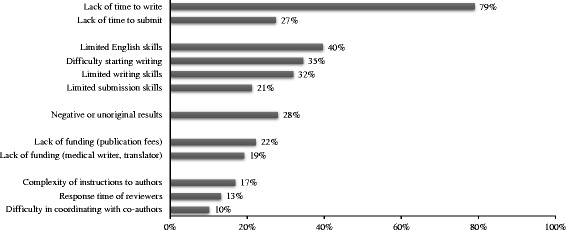
Barriers to publication according to respondents (%). *N* = 1191; more than 1 answer possible. Answer choices are grouped depending on the barriers to publication: i.e., lack of time, limited skills, study results, lack of funding, or other barriers

According to the respondents (*N* = 1191), the main barriers to publication were lack of time to write articles (79%), limited skills in English (40%), limited skills in writing (32%) and difficulty in starting writing (35%) (Fig. 2). Female gender and working as hospital practitioner or senior registrar/hospital university assistant were significantly associated with self-reported limited skills in English (*P* = 0.021 and *P* < 0.001, respectively), self-reported limited skills in writing (*P* < 0.001), and with difficulty in starting writing (*P* < 0.001). Young age was significantly associated with self-reported limited skills in writing (*P* < 0.001), but not with self-reported limited skills in English (*P* = 0.55) or difficulty in starting writing (*P* = 0.2). Compared with other respondents, respondents reporting limited-skills in English, limited skills in writing, or difficulty in starting writing were less frequently first, second/third, or last author of their publications (*P* < 0.001) and less frequently satisfied with their number of publications (*P* < 0.001); they more frequently said that they would appreciate technical support for future publication (*P* < 0.001). Respondents with self-reported limited skills in English (*P* = 0.013) more frequently received support for their previous publications than the other respondents. On the contrary, no significant difference in support was observed between respondents with and without self-reported limited skills in writing (*P* = 0.35) or difficulty in starting writing (*P* = 0.32). Factors associated with self-reported limited skills in writing are presented in Table 2.

**Table 2:** Factors associated with self-reported limited skills in writing (*N* = 1191)

	Limited skills in writing^a^		Chi *P-value*
	No (*N* = 811)	Yes (*N* = 380)	
Gender: female	48%	68%	<0.001^1^
Age (years)			<0.001^2^
< 35	20%	41%	
35–45	35%	38%	
45–55	25%	17%	
55–65	17%	4%	
> 65	2%	1%	
Position			<0.001^1^
Hospital practitioner	18%	28%	
Senior/hospital university assistant	46%	67%	
University professor/hospital practitioner	15%	4%	
University assistant professor/hospital practitioner	21%	1%	
First author^b^ in:			<0.001^2^
0 article	27%	52%	
1–2 articles	40%	40%	
3–4 articles	22%	7%	
5 and + articles	10%	1%	
Second or third author^b^ in:			<0.001^2^
0 article	20%	38%	
1–2 articles	42%	46%	
3–4 articles	25%	14%	
5 and + articles	13%	2%	
Last author^b^ in:			<0.001^2^
0 article	50%	86%	
1–2 articles	27%	11%	
3–4 articles	12%	2%	
5 and + articles	11%	0%	
Not publishing enough	69%	93%	<0.001^1^
No external support for previous articles^b^	73%	70%	0.35^1^
Limited skills in English	30%	60%	<0.001^1^
Difficulty in starting writing	24%	56%	<0.001^1^
Would appreciate technical support for a future publication	83%	96%	<0.001^1^

^a^Rounded values; ^b^In the previous 2 years

Tests used: ^1^Pearson chi-square test, ^2^Proportional odds likelihood ratio chi-square test

### Motivation and satisfaction

**Fig. 3:**
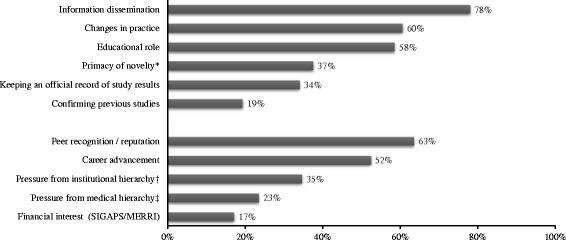
Respondents’ main reasons for publishing (%). *N* = 1191; more than 1 answer possible. Answer choices are grouped depending on the reasons for publishing: i.e., scientific objectives versus author’s career or research funding. * Being the first to publish; † AP-HP, University…; ‡ Medical supervisor in the department

According to the respondents, the main reasons for publishing were scientific (dissemination of the results and change in practice rather than being the first to publish) and for peer recognition and career advancement (Fig. 3). Although 35% of respondents reported publishing under pressure from their institutional hierarchy, only 17% reported publishing to obtain SIGAPS points.

Seventy six percent of authors claimed they did not publish enough, mainly because of lack of time (85%). Seventy four percent estimated that writing these recent articles (from drafting the manuscript to final acceptance by the journal) took between 6 months and 2 years.

The main reasons for refusal of articles was, according to the respondents: editorial judgment of lack of originality (41%) or problems of methodology and/or results (32%), negative and inappropriate comments by reviewers (25%), poor choice of the journal by the authors (21%), and the fact that study results were of purely French origin (15%). Respectively 19% and 11% of respondents claimed that refusal was due to poor English or writing quality. Conflicts of interest were rarely mentioned (4.6%).

### Perceived needs

Most respondents (87%), and in particular those who published less than 3 articles in the previous 2 years, regardless of their position (i.e., first, second or third, or last authors), would accept technical support to overcome their difficulties in publishing. They would particularly appreciate support for English (79%) or critical reediting (63%), formatting before article submission (52%), writing responses to reviewers (47%), writing some parts of the article (41%), and/or article submission (44%). This support was expected to save time (92%), avoid some refusals (83%), or increase high-impact-factor journal submission and acceptance (75%).

**Fig. 4:**
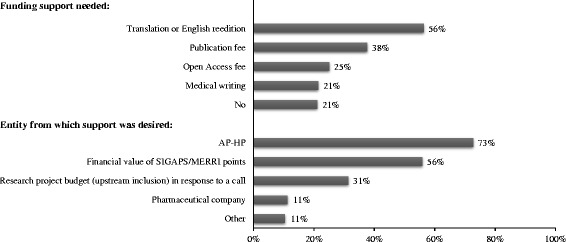
Funding support needed and expected financial support according to respondents (%). *N* = 1191; more than 1 answer possible. Answer choices to the following question are grouped under “Funding support needed”: “*For your forthcoming article, do you need (or do you think needing) funding support for…?*”. Answer choices to the following question are grouped under “Entity from which support was desired”: “*According to you, how could this support be funded?*”

For future articles, 79% of respondents claimed that they would need funding, in particular for English reediting (56%), publication (38%), Open Access (25%), or medical writer (21%) fees. According to respondents, this financial support should be covered by their institution, AP-HP, (73%), the added financial value obtained by their department from previous research activity and publications (SIGAPS/MERRI points; 56%), the upstream research project budgeting in response to a call for projects (31%), or a pharmaceutical company (11%) (Fig. 4).

### Free comments

Free comments were made by 256 (21%) respondents. Overall, these comments showed respondents’ dissatisfaction and/or referred back to issues raised by this questionnaire. Some barriers to publication already dealt within the questionnaire were reiterated: lack of time (27%, mostly due to clinical and administrative overload), limited skills in English (11%), and lack of funding (6%). Beyond the barriers to publication evaluated by the questionnaire, other upstream barriers which obviously impact the publication process emerged: lack of assistance for statistical analyses (19%), complexity of regulatory dossier preparation before study implementation (7%), lack of assistance in information technology (database management, supply of software such as for managing bibliographies and references) (6%), and lack of logistical assistance in the conduct of studies (e.g., clinical research technician, data collection) (6%).

A few respondents commented that some topics are difficult to publish in high impact journals (e.g., palliative care, qualitative research, pediatric surgery, environmental pathology). Some also raised the growing issue of conflicts of interest of reviewers in the refusal of articles and believed that being a non-English speaking author reduced their chances. Of the respondents who made a free comment, 18 (7%) regretted lack of information about the use of SIGAPS and that the financial value of SIGAPS/MERRI points was not allocated back to researchers (a point sometimes vehemently directed at their institution). Finally, 47 (18%) of respondents spontaneously welcomed the survey (only 1 respondent commented that the survey had no interest, but still answered all items).

## Discussion

DIAzePAM is a survey assessing potential difficulties experienced by French researchers when preparing articles for biomedical journals. It also assessed their needs for future support. The number of respondents was high, significantly higher than expected, given that the topic of the survey did not focus on a strictly salient medical issue, and higher than usually observed for similar surveys with comparable topic [25, 26].

### What are the difficulties?

Lack of time was the main barrier to publication. This result was consistent with the literature [27] and with the number of respondents who had published but thought they were not publishing enough (76% of authors), mainly because of lack of time (85%). It was also consistent with the percentage of authors claiming to have had difficulty in writing the Discussion (60%), as this section needs time to think, read the literature, and compare present and previously published results [28, 29].

The other main reasons given by more than 20% of respondents included self-reported limited skills in English or in writing, limited submission skills, and difficulty in starting writing. As expected, being young and working as hospital practitioner or senior registrar/hospital university assistant were associated with self-reported limited writing skills, but being young was not associated with self-limited skills in English. Surprisingly, being a woman was a factor associated with self-reported limited skills in writing. This possibly adds up with the other reasons leading female physicians to have a lower publishing rate than their male colleagues [30]. Unfortunately, combination of lack of time and limited skills increase difficulties. Indeed, English and writing skills make writing easier; easy writing takes less time and energy, and this decreases the risk of procrastination. It has been showed that this combination decreased the chance of publishing in a high-impact journal: respectively 78% and 70% of researchers publishing in high-impact journals had English as current official work language and spent more than 50% of their time on research versus for 35% and 27% researchers publishing in low-impact journals [31].

According to the respondents, another significant barrier to publication is negative or unoriginal results. Negative results are more difficult to publish, as there is a greater interest in publishing results that can immediately change clinical practice [32]. However, there is presently an international groundswell in favor of proper transparency and full reporting of all results so as to avoid the pitfalls of publication bias, and this movement involves different stakeholders such as Drug Agencies [33] and medical journals (www.alltrials.net), so that this barrier should now be a thing of the past. Knowledge of negative results may be as useful as positive results in achieving good non-deleterious patient care [34].

Last but not least, lack of funding, in particular for publication fees, was a barrier to publication for 22% of respondents. Over the past decade, the budget for publications has become a growing problem, in particular with the increasing number of Open Access journals that allow free access to readers, but not authors [35, 36]. In addition, 19% of respondents declared that they lacked funding for a medical writer or translator. According to Pavia et al., publishing in high- and low-impact journals is also dependent of financial resources [31].

### What room is there for improvement?

On the one hand, most respondents (87%) would accept technical support to overcome their difficulties in publishing, calling into question the legend of the researchers who must do everything (research and writing) themselves [37]. On the other hand, among the 28% of authors who already called upon additional support, only 26% had used services of a medical writer. This is possibly due to the lack of funding. However, as 61% of authors who already called upon additional support had used services of a translator, another possible reason is the lack of knowledge of the job of medical writers and the confusion between the jobs of translator and medical writers. Indeed, English reediting, critical reediting, formatting/submitting and writing were the main tasks for which support was needed, and medical writers fulfill all these functions. One thing is to be a fluent writer of English, and quite another to know the socio-pragmatic features of today’s academic rhetoric. Gattrell et al. [38] recently showed that declared professional medical writing support was associated with more complete reporting of clinical results and higher quality of written English in the sample of journals they analyzed. In addition, in this study, authors who called upon additional support more frequently published at least once as first author in the previous 2 years than the other authors, suggesting that external support increased publication as first author. Thus, as is the case in Scandinavian countries, French universities and/or research centers should have an “academic/scientific writing center” with competent applied linguists well versed in the rhetoric of today’s academic/scientific discourse. Such a Center should be free of charge for all researchers. Introduction of publications officers, regular and early training in English and writing [39], and use of Writing Aid Tool (WAT) such as the online Consort-based Web tool for randomized controlled trials [40] could contribute to increase both number and value of published research articles.

In addition to technical support, 79% of respondents declared a need for funding support. Respondents thought that support could be provided by AP-HP directly (73%), or through the added financial value obtained by their department from previous publications (SIGAPS/MERRI points; 56%). In 18 (7%) free comments, respondents regretted that the financial value of SIGAPS/MERRI points was not currently clearly allocated back to researchers by AP-HP, and a lack of information about its use. Such funding support could allow researchers to pay for English reediting, publication, Open Access, or medical writers’ fees. These results suggest that the process of conversion between one type of capital (credibility) and another (material resources), known as “cycles of credit” [41], was perceived as damaged and needs to be reactivated. According to this process (and as expected by respondents), articles issued from arguments elaborated using research data contribute to increase recognition which in turn increases funding enabling investment in human and material resources necessary for more data collection and arguments constructions.

### What are the strengths and limitations of the present survey?

Electronic surveys avoid the need for double entry and enable questionnaire personalization (questions are automatically hidden or revealed according to previous answers) and timeliness, at low cost [42, 43]. Limited access to the Internet, overloaded e-mail inboxes and obsolete e-mail addresses are limitations to electronic surveys, and were encountered in the present study; besides overloaded inboxes and obsolete e-mail addresses, some researchers registered in the AP-HP SIGAPS database contacted us for help, finding it impossible to connect due to their institution’s firewalls. Nevertheless, as the target participants were pressed for time, the method was probably the best way to conduct the present study. Other limitations common to all questionnaires, and not specific to electronic surveys, are questionnaire size (long questionnaires are often less filled out than short ones), user-friendliness, and type of response. We paid careful attention to completion time and type of response in developing the DIAzePAM questionnaire, and ran some tests on the draft version. However, we did not include any questions on user-friendliness and so cannot know whether the questionnaire was well perceived and easy to complete.

Only volunteers responded to the questionnaire, automatically creating a bias. As no compensation for participation was offered, it can be hypothesized that researchers who perceived difficulties in preparing manuscripts for publication may have been incentivized to respond to the online survey. However, some respondents had published several articles in the previous 2 years. The decision to respond to the online survey or not may also have been affected by the fact that the survey was conducted in the name of the AP-HP. Sociodemographic data on AP-HP staff are sparse and do not target the population of researchers who publish or intend to publish, hindering comparison with the respondents to the present survey. However, based on figures for medical staff working at AP-HP in 2014 [44], 25% of assistant professors, 20% of senior registrars, 15% of professors and 6% of hospital practitioners responded to this online survey. Although hospitals practitioners were under-represented as compared with the other professionals, this result is probably consistent with the population of medical staff performing research and publishing.

## Conclusions

Most of the 1191 researchers working in AP-HP (France) who responded to the DIAzePAM survey experienced difficulties with publication and would appreciate technical and financial support in the future. These results raise the question of how to improve the number and quality of publications in biomedical journals by researchers in AP-HP (and, beyond Paris, in France) and point to some potential solutions: e.g., improving English and writing skills of researchers, funding external medical writers or creating an internal structure to assist researchers with publication, allocating back the financial value of SIGAPS/MERRI points for research and publication.

## Additional file

Additional file 1: English version of the DIAzePAM questionnaire. English version of list of items analyzed in the present article. (PDF 169 kb)

## Abbreviations

AP-HP: *Assistance Publique – Hôpitaux de Paris*
DIAzePAM: *Difficultés des Auteurs à la Publication d’Articles Médicaux*
MERRI: *Missions d’Enseignement, de Recherche, de Référence et d’Innovation*
SIGAPS: *Système d’Interrogation, de Gestion et d’Analyse des Publications Scientifiques*
