# Supplementary material for: Barriers to publishing in biomedical journals perceived by a sample of French researchers: results of the DIAzePAM study
Source: BMC Med Res Methodol. 2017 Jul 10;17:96. doi: 10.1186/s12874-017-0371-z (PMC5504731; doi:10.1186/s12874-017-0371-z)
Supplement: Additional file 1: — English version of the DIAzePAM questionnaire. English version of list of items analyzed in the present article. (PDF 169 kb) [file 12874_2017_371_MOESM1_ESM.pdf]

Additional file 1: English version of the DIAzePAM questionnaire.

Q01. Over the last 2 years, how many times did you appear as…?
(more than 1 answer possible, only 1 per SQ)
SQ001. 1st author in
- ☐ 0 article
- ☐ 1-2 articles
- ☐ 3-5 articles
- ☐ > 5 articles

SQ002. Last author in
- ☐ 0 article
- ☐ 1-2 articles
- ☐ 3-5 articles
- ☐ > 5 articles

SQ004. 2nd, 3rd or penultimate author in
- ☐ 0 article
- ☐ 1-2 articles
- ☐ 3-5 articles
- ☐ > 5 articles

SQ005. Other rank among authors
- ☐ 0 article
- ☐ 1-2 articles
- ☐ 3-5 articles
- ☐ > 5 articles

Q01a. How would you describe your perception concerning your publications?
(only 1 answer possible)
- ☐ I publish enough
- ☐ I don’t publish enough
- ☐ No answer

Q01b. Why?
(more than 1 answer possible)
- ☐ I don’t do enough research
- ☐ I’m not in a stimulating environment (no publishing or research activities in the department)
- ☐ I’m not a member of a research team
- ☐ I don’t have time
- ☐ I don’t need it
- ☐ I am not interested
- ☐ Other

Q02. What was your real writing contribution in the last 3 published article(s)?
(more than 1 answer possible)
- ☐ Study design and development
- ☐ Data analysis and interpretation
- ☐ Writing the manuscript
- ☐ Critical review of the manuscript
- ☐ Approval of the final version of the manuscript
- ☐ Journal selection
- ☐ Manuscript submission to the journal
- ☐ Answers to reviewers, manuscript resubmission(s)
- ☐ Minimal
- ☐ Investigator (I included patients but did not contribute in manuscript writing)

Q03. According to you, what was the mean writing duration of this or these articles (from the beginning of the writing process to final acceptance by the journal)?
(only 1 answer possible)
- ☐ 0 to 6 months
- ☐ 6 to 12 months
- ☐ 1 to 2 years

- ☐ 2 to 3 years
- ☐ > 3 years
- ☐ I don’t know

Q04. According to you, which parts were the most difficult to write?
(more than 1 answer possible)

- ☐ Introduction
- ☐ Material and methods
- ☐ Results
- ☐ Tables and figures
- ☐ Discussion
- ☐ All
- ☐ Neither of them
- ☐ I don’t know

Q05. Did you call upon additional support, apart from authors, for writing one of these articles?
(only 1 answer possible)

- ☐ Yes
- ☐ No

Q05a. Support came from
(more than 1 answer possible)

- ☐ One or more non-author colleague(s)
- ☐ English-speaking relationship(s)/translator(s)
- ☐ Medical writer(s)
- ☐ I don’t know exactly

Q05b. The person who helped was
(more than 1 answer possible)

- ☐ Paid
- ☐ Unpaid
- ☐ Cited in the Acknowledgements section of the article
- ☐ Cited among authors
- ☐ None of this
- ☐ I don’t know

Q06. For your articles, what are your journal selection criteria?
(more than 1 answer possible)

- ☐ Acceptance rate
- ☐ Impact Factor
- ☐ Publication speed
- ☐ Degree of specialization
- ☐ Topic already accepted by the journal
- ☐ High rank in SIGAPS classification of journals
- ☐ Open Access journal
- ☐ Acquaintance with of one of the editors
- ☐ Opportunity to suggest reviewers

Q07. In general, which journal model do you prefer?
(only 1 answer possible)

- ☐ Open access (free PDF of the article)
- ☐ Classic model (access to the article after subscription to the journal)
- ☐ No preference

Q08. Which barriers do you encounter to publish your results?
(more than 1 answer possible)

- ☐ Negative or unoriginal results
- ☐ Limited writing skills
- ☐ Difficulty starting writing
- ☐ Lack of time to write

- Complexity of instructions to authors
- Limited English skills
- Difficulty in coordinating with co-authors
- Lack of time to submit
- Limited submission skills
- Lack of funding (publication fees)
- Lack of funding (medical writer, translator)
- Response time of reviewers
- Lack of funding
- Other

Q09. According to you, what are the main reasons for editor's refusal?
(more than 1 answer possible)
- Lack of originality
- Problems of methodology and/or results
- Purely French origin (anglophone journal)
- Poor English quality
- Poor writing quality
- Erroneous or inappropriate comments by the reviewers chosen by the journal
- Poor choice of the journal (according to the article topic)
- Authors' conflicts of interest
- I don't know

Q10. Would you accept technical support to overcome your difficulties in publishing?
(only 1 answer possible)
- Yes
- No

Q10b. If yes, for which tasks would you like support?
(type 0 no need, 1 possibly, 2 need, to 3 important need)

SQ001. Writing some parts of the article
- 0: No need
- 1: Possibly
- 2: Need
- 3: Important need

SQ002. Tables and figures
- 0: No need
- 1: Possibly
- 2: Need
- 3: Important need

SQ003. English reediting
- 0: No need
- 1: Possibly
- 2: Need
- 3: Important need

SQ004. Critical reediting
- 0: No need
- 1: Possibly
- 2: Need
- 3: Important need

SQ005. Formatting before article submission
- 0: No need
- 1: Possibly
- 2: Need
- 3: Important need

SQ006. Management of co-authors comments
- 0: No need
- 1: Possibly
- 2: Need

- 3: Important need

SQ007. Article submission
- 0: No need
- 1: Possibly
- 2: Need
- 3: Important need

SQ008. Writing responses to reviewers
- 0: No need
- 1: Possibly
- 2: Need
- 3: Important need

SQ009. Other
- 0: No need
- 1: Possibly
- 2: Need
- 3: Important need

This support would enable you to

Q10d. Save time

(only 1 answer possible)
- Yes
- No
- No answer

Q10e. If yes

(only 1 answer possible)
- 0-3 months
- 3-6 months
- 6-9 months
- 9-12 months
- > 12 months

Q10f. Increase high impact factor journal submission

(only 1 answer possible)
- Yes
- No
- No answer

Q10g. Avoid some refusals

(only 1 answer possible)
- Yes
- No
- No answer

Q11. For your forthcoming article, do you need (or do you think needing) funding support for...?

(more than 1 answer possible)
- Medical writing
- Translation or English reedition
- Publication fee
- Open Access fee
- No need

Q12. According to you, how could this support be funded?

(more than 1 answer possible)
- Research project budget (upstream inclusion) in response to a call
- AP-HP
- Financial value of SIGAPS/MERRI points
- Pharmaceutical company
- Other

Q13. Why do you want to publish ?
(more than 1 answer possible)

- ☐ Primacy of novelty (SQ001)
- ☐ Educational role (SQ002)
- ☐ Changes in practice (SQ003)
- ☐ Keeping an official record of study results (SQ004)
- ☐ Peer recognition / reputation (SQ005)
- ☐ Career advancement (SQ006)
- ☐ Financial interest (SIGAPS/MERRI) (SQ007)
- ☐ Pressure from medical hierarchy (SQ008)
- ☐ Information dissemination (SQ009)
- ☐ Confirming previous studies (SQ010)
- ☐ Pressure from institutional hierarchy (AP-HP, university...) (SQ011)

Q14a. Sex
(only 1 answer possible)

- ☐ Male
- ☐ Female

Q14b. Age (years)
(only 1 answer possible)

- ☐ < 35
- ☐ 35-45
- ☐ 45-55
- ☐ 55-65
- ☐ > 65

Q14c. Profession
(only 1 answer possible)

- ☐ Physician
- ☐ Pharmacist
- ☐ Odontologist
- ☐ Biologist
- ☐ Geneticist
- ☐ Other

Q14d. Position
(only 1 answer possible)

- ☐ Hospital practitioner
- ☐ Senior registrar, hospital university assistant
- ☐ University professor/hospital practitioner
- ☐ University assistant professor/hospital practitioner
- ☐ Other

Q14e. Do you hold the following degrees?
(more than 1 answer possible)

- ☐ PhD
- ☐ Accreditation to direct research
- ☐ Neither of the two

Q15. Do you want to add a comment?
(free)
